# Kreosote in Hæmatemesis—Report of a Case

**Published:** 1839-08-10

**Authors:** Isaac Parrish


ই# MEDICAL EXAMINER.

DEVOTED TO MEDICINE, SURGERY, AND THE COLLATERAL SCIENCES.

No. 32.] PHILADELPHIA, SATURDAY, AUGUST 10, 1839. [Vol. II.

### *Kreosote in Hæmatemesis—Report of a Case*, by Isaac Parrish, M. D.

The powerful styptic properties of kreosote, have been so repeatedly tested by recent observations, that it has obtained, by common consent, a prominent place amongst this class of remedial agents. In hæmorrhages occurring in individuals of enfeebled constitution, and arising from an atonic condition of the vessels of a part, its powers, both as a stimulant and a styptic, would seem to indicate it as a peculiarly effective remedy. This form of disease is presented to us in the hæmorrhage from the stomach, which sometimes occurs in persons addicted to the use of ardent spirits, and it is to such a case that I would direct attention by the following communication.

*Seventh month* 17*th*, 1839.—I was called about 6 P. M., to see J. C., a tavern-keeper, of about forty-five years of age, a remarkably stout, athletic man, accustomed to the free use of spirituous liquors, and addicted to the pleasures of the table. I had attended J. C. on a former occasion, about two years since, with an attack of hæmatemesis, which was of a moderate character, and was speedily controlled by remedies. I then informed him of the danger of a recurrence of the disease, if he persisted in his dissipated habits,—and my caution produced, for a time, a salutary change. He had, however, returned to his old ways, and I was now called to visit him for the same disease in a more violent form. The attack had commenced in the early part of the afternoon, and he had twice vomited a considerable quantity of blood, mixed with the contents of the stomach. I found that on the preceding day he had been indulging more freely than usual in his potations, and that he had been complaining for some days previously of a numbness and sense of fulness in the back of his neck and head, with loss of appetite, and uneasiness about the stomach. I directed him to go to bed, and keep perfectly quiet, take iced lemonade for drink, and have eight cups applied to the epigastrium, with the following mixture:—℞. Acid Nitrosi gtt. viij., Aquâ Camphor. ℥iv. To take a tablespoonful every hour, diluted.

I was summoned in haste to see my patient about midnight, and found him pale, and exhausted, with a cool skin, and feeble pulse. He had just had a copious discharge of pure blood from the stomach—I should suppose, about a pint. He complained of a sense of weight at the epigastrium, preceding the discharge, which was relieved by the vomiting. He was incessantly calling for drink. I directed ice to be held in the mouth, and discouraged the use of large draughts of water, to suspend the acid drink, and take a pill every hour of Acet. Plumbi gr. ij., Pulv. Gum Kino gr. j., Pulv. Opii gr. ¼.

18*th*.—I was called to him at 7 o'clock, A. M. He had vomited small quantities of blood several times during the night, and had had a copious discharge just before I arrived, besides two stools containing dark blood. His pulse was now irregular and feeble; skin cold, pallid, and covered with a clammy sweat. He had slept very little during the night. The pills appeared to sicken him, and he had taken but two. At my request, a consultation was called, and Dr. Otto visited him with me. The pills were discontinued, and we placed him on the muriated tincture of iron, gtt. x. every hour. Ice and cold drinks continued, and an anodyne enema; sinapisms to the extremities. We met again at noon, and found our patient still extremely feeble; he had vomited several times a small quantity of blood. We concluded, if it should return in large quantities, to try the kreosote. I saw him again at 4 o'clock. He had just vomited a large quantity—I should suppose, a pint—of dark blood, containing coagula. His pulse was extremely feeble, and skin as cold as marble. He had settled his affairs, and looked forward to death as inevitable. I directed a large blister to be placed over the abdomen, sinapisms to the extremities to be renewed, and take a dessert-spoonful of the following mixture every hour:—Kreosote gtt. viij., Pulv. Gum Arab., Pulv. Sacch. Alb. āā ʒj., Aquâ ℥ij.

9 *P. M.*—Had no return of the bloody vomiting since taking the kreosote. He had vomited once after the second dose, but merely threw up the fluids from the stomach, without any admixture of blood; the odour of the kreosote was very perceptible in the fluids discharged. Slight reaction had occurred, with an evident tendency to delirium. The kreosote was directed to be continued through the night every two hours, with black drop gtt. x. every two hours.

19*th*.—Pulse improved; skin warm; blister has drawn freely; patient dozed at intervals during the night; talked wildly on waking, but was rational when roused; desires food; no return of vomiting. He was allowed nutritious drinks in small quantities, frequently repeated, and his medicines were given at longer intervals. He continued to improve from this time, and at the end of ten days was able to move about his house.

In this case, the kreosote certainly produced a prompt and powerful impression, and apparently rescued the patient from death, inasmuch as the remedies generally employed in such cases failed to produce any effect.

